# Low Vitamin D Levels at Birth and Early Respiratory Outcome in Infants With Gestational Age Less Than 29 Weeks

**DOI:** 10.3389/fped.2021.790839

**Published:** 2022-01-21

**Authors:** Honoré Papalia, Anais Samonini, Christophe Buffat, E. Gras, Clotilde des Robert, Jean-Francois Landrier, Vanessa Pauly, Farid Boubred

**Affiliations:** ^1^Neonatal Unit, Hospital University La Conception, APHM, Marseille, France; ^2^Laboratory of Biochemistry and Molecular Biology, Hospital University la Conception, APHM, Marseille, France; ^3^Aix-Marseille University, INSERM, INRAE, C2VN, Marseille, France; ^4^EA 3279, CEReSS—Health Service Research and Quality of Life Center, Public Health and Medical Information Department, APHM, Marseille, France

**Keywords:** extremely preterm infants, RDS—respiratory distress syndrome, surfactant, vitamin D, bronchopulmonary dysplasia, LISA (less invasive surfactant administration), nasal CPAP (continuous positive airway pressure), SGA (samll for gestational age)

## Abstract

**Background:**

Vitamin D (VitD) is involved in lung development but its influence on respiratory distress syndrome of extremely preterm (EPT) infants have been little investigated. In this study, we examined the influence of low vitamin D status at birth on early respiratory outcomes of this vulnerable infant population.

**Methods:**

Cord blood 25(OH)D levels ≤ 75 nmol/L were considered as Low vitamin D levels. Stepwise logistic regression and classification regression-tree analyses were used and the primary outcome was the combined outcome of death or mechanical ventilation need by the end of the first week (death or MV DoL7) as a marker od RDS severity.

**Results:**

The mean (SD) GA and birth weight were 26 (1.4) weeks and 801 (212) gr, respectively; 81/109 (74%) infants had low 25(OH)D levels. Infants with low VitD levels had 25% higher initial FiO_2_ levels (*p* < 0.05) and were more likely to be mechanically ventilated on DoL7 (36 vs. 7%, *p* < 0.05). Adjusted for gestational age, they had 10-fold higher odds of death or MV DoL7 (*p* < 0.01). By regression tree analysis, the rate of death or MV DoL7 increased from 18 to 71% in infants with GA < 26 weeks and with cord blood 25(OH)D levels higher and lower than 74 nmol/L, respectively (*p* < 0.05).

**Conclusion:**

Low vitamin D levels at birth are associated with early adverse respiratory outcomes in infants with GA less 29 weeks. Further largest studies are needed to confirm this association.

## Introduction

Respiratory distress syndrome (RDS) can be considered as a developmental disease which predominantly concerned extremely preterm (EPT) infants. These infants often have severe form of RDS with early high oxygen needs, multiple surfactant administration and prolonged mechanical ventilation. Early markers of RDS severity may help to better identify high risk infants and provide optimal care to prevent short and long term RDS complications such as bronchopulmonary dysplasia (BPD).

Vitamin D is well known for its major role in fetal bone metabolism, but recent evidence also shows a biological role in normal lung development and in surfactant synthesis (1–4). In animals, maternal gestational vitamin D depletion affects fetal lung development with reduced alveolar numbers and impaired bronchial structure (2, 4). Children born to vitamin D-deficient mothers are more likely to have respiratory symptoms such as wheezing events, asthma, respiratory tract infections or lung dysfunction (5–9). In premature infants, with immature lungs, the optimal dose of vitamin D supply is still under investigation. The few randomized clinical studies looking into the effects of vitamin D supply on respiratory outcomes have shown variable effects with either no effects or decreased RDS severity (10–12).

Preterm infants are at high risk of vitamin D deficiency at birth, with observational studies showing a prevalence as high as 90% (13–15). A part of that deficient state may result from limited vitamin D transfer from the mother. Indeed, the fetus entirely depends on the maternal vitamin D status, and placental transfer mainly occurs in the third trimester (16). Furthermore, the leading obstetrical causes of prematurity, including preeclampsia, gestational diabetes, small for gestational age (SGA) and threatened preterm delivery, are often associated with maternal vitamin D deficiency (17–19). However, the adverse effects of maternal/fetal vitamin D deficiency on neonatal outcomes remain conflicting in very preterm infants and still need to be further investigated in extremely preterm (EPT) infants, in particular.

The objective of the study was to examine the association between vitamin D status at birth and early respiratory outcomes of infants with GA <29 weeks.

## Methods

This observational and retrospective study included preterm infants born with GA between 24 weeks^+0^ and 28 weeks^+6^ between November 1, 2018 and April 31, 2020 that were admitted to the Neonatal Intensive Care Unit (NICU), Hospital University La Conception, Marseilles, France. This study was part of a quality improvement initiative aimed at optimizing vitamin D supplementation in preterm infants. All infants followed the same postnatal vitamin D supply protocol during the study period; no changes have been made between the two groups. Blood 25 hydroxyvitamin D [25(OH)D] concentration was measured at birth from umbilical cord blood. This study was approved by local ethical and privacy health data protection committees (N°. DSN_2019-06-20_7706). Clinical and biological data collection were anonymized. Parents were informed of the study and were not required to sign informed consent, but they could oppose data collection. For this study, preterm infants born at our institution and with specifically cord blood 25(OH)D measurements were eligible; infants with congenital or genetic abnormalities or whose parents refused to participate in the study were excluded. Cord blood 25(OH)D levels were determined by chemiluminescence (Roche Diagnostics, Mannheim, Germany). This method has been calibrated to liquid chromatography–mass spectrometry. In preterm infants, the optimal 25(OH)D levels for adequate respiratory outcome are unknown. We used the Endocrine Society recommendations for defining vitamin D status that are made for healthy bone growth (20). For this study, two groups of infants were compared: infants with cord blood 25(OH)D levels ≤ 75 nmol/L (LowVitD status group) and infants with cord blood 25(OH)D levels >75 nmol/L (SufficientVitD status).

### Early Respiratory Outcome

The primary outcome was the combined outcome of death in the first week of life or the need for mechanical ventilation (MV) on day of life (DoL) 7 (death or MV DoL7). We also compared other respiratory outcomes, such as delivery room intubation within 15 mins of life (MV DR), need for MV on DoL3 (MV DoL3) and 7 (MV DoL7) and any need for MV in the first week of life (MV W1). Respiratory management was standardized; all spontaneously breathing infants were initially managed with nasal continuous positive airway pressure (NCPAP). Surfactant was indicated when the fractional inspired oxygen concentration (FiO_2_) reached 30% or when the infant required mechanical ventilation (21, 22). Observational studies have confirmed that FiO_2_ exceeding 0.30 in infants initially managed on NCPAP for RDS predicted NCPAP failure (22). Timing and FiO_2_ levels immediately before surfactant administration were collected. The less invasive surfactant administration (LISA) technique or surfactant administration after a brief intubation procedure (intubation, surfactant and extubation, INSURE procedure) were compared between the two groups.

### Data Collection and Statistical Analysis

Maternal and neonatal outcomes were collected from infant medical charts. Gestational age (GA) was calculated from first trimester obstetrical ultrasound, and small for gestational age (SGA) was determined using Fenton growth charts (23). At birth, umbilical cord milking or delayed cord clamping for at least 30 s defined placental transfusion. Severe cerebral injury included stage 3 and 4 intraventricular hemorrhage (Papile's classification), periventricular leukomalacia and isolated ventricular dilatation at term. The variable of severe digestive disease included necrotizing enterocolitis (NEC) stage 2 or more (Bell's classification) and isolated intestinal perforation. Late onset sepsis after DoL3 and severe retinopathy (stage 3 and more) were noted as well. Bronchopulmonary dysplasia (BPD) was defined as the need for oxygen supplementation or respiratory support at 36 weeks postmenstrual age. We used Jensen et al.'s definition to classify BPD severity: infants who required nasal cannula flow rate >2 L/min, non-invasive positive airway pressure, or invasive mechanical ventilation were classified as grade 2–3 BPD (24).

To compare both infant vitamin D status groups, we performed univariate statistical tests: Chi-squared (or Fischer's exact tests) and Student's *t* tests (or Mann-Whitney test) were used as appropriate to compare groups for categorical and quantitative variables, respectively. All statistical tests were two-sided. To analyze factors associated with the combined variable of death or MV DOL7, we performed univariate and multivariate logistic regression analyses. We included variables with *p* <0.20 in univariate analyses into a step-by-step backward multivariable analysis. We also used Firth's penalized estimation using logistic regression to account for the low number, which led to imprecisions in parameter estimation. The results are presented as odds ratios (ORs) and their 95% confidence intervals (CIs).

Then, as an explanatory analysis, we performed a classification regression tree analysis (CART) to identify an easy-to-understand algorithm for predicting the primary outcome of death or MV at DoL7. This analysis allows automatic selection of categorical variables and of optimal cutoff points of continued variables and generates a classification tree when the more important variable in relation to outcome is at the top of the decision tree (25). Variables initially entered for consideration into the CART analysis were the following: GA, sex, multiple pregnancies, preterm premature rupture of membranes, chorioamnionitis, antenatal corticosteroid therapy, cesarean section, placental transfusion and cord blood 25(OH)D levels. This CART analysis was mainly performed to reveal an optimal threshold for blood 25(OH)D levels. Due to the low number of subjects involved in the analysis, it was performed on the whole dataset; the accuracy was tested using three-fold cross validation repeated 200 times. The analyses were performed using IBM SPSS Statistics for Windows (IBM Company, Chicago, IL, USA), Version 20.0, and SAS®, Version 9.4 (SAS Institute Inc, Cary, NC, USA). In this study, *p* < 0.05 were considered statistically significant. The CART tree was generated using the RPART package in R® Software.

## Results

During the study period, 112 infants met the eligibility criteria, and 3 infants were excluded: 2 for congenital and genetic anomalies and 1 for parental refusal (Supplementary Figure). The mean (SD) GA and birth weight were 26 (1.4) weeks and 801 (212) gr, respectively. The mean (SD) cord blood 25(OH)D level was 53 (32) nmol/L. A total of 81 (74%) infants had low cord blood 25(OH)D levels (≤ 75 nmol/L) and 56 (51%) infants had cord blood 25(OH)D levels <50 nmol/L.

**Figure 1:**
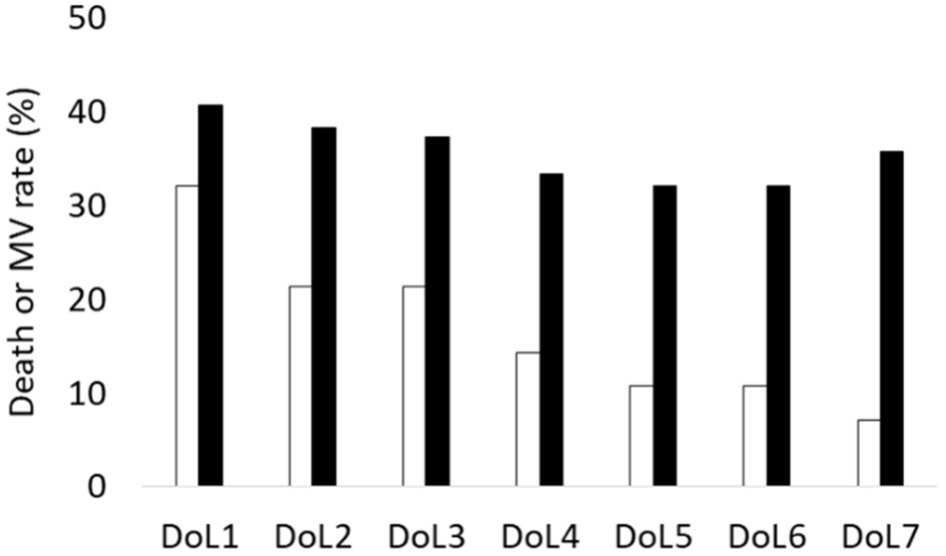
Daily combined death or mechanical ventilation (death or MV) rate in the first week of life between LowVitD status and SufficientVitD status infants. During the first week of life, the rate of the combined outcome of death or MV need were high in infants with low vitamin D levels < 75 nmol/L (black bars).

Obstetrical and neonatal characteristics, in particular GA, birth weight, SGA, sex ratio and exposure to antenatal corticosteroids, were comparable between infants with low and sufficient cord blood 25(OH)D levels (Table 1). However, early respiratory outcomes differed between the two groups of infants (Table 2). The timing, dose number and technique procedure (LISA or INSURE) of surfactant administration were not different, but FiO_2_ levels at the time of surfactant administration were 25% higher in LowVitD status infants (*p* = 0.02). At DoL7, the rate of the combined outcome of death or MV requirement was higher in LowVitD status infants (36 vs. 7%, *p* < 0.01). These infants were also more likely to receive MV during the first week (MV W1) (53 vs. 32%, *p* = 0.05) than the group of SufficientVitD status infants (Figure 1) and tended to be more frequently ventilated by high frequency oscillation (46 vs. 11%, *p* = 0.06). At DoL7, 93% of infants who died or needed MV were from the LowVitD status group.

**Table 1:** Obstetrical and neonatal characteristics according to vitamin D status at birth.

**Characteristics, n (%)**	**All** **population** **(*N* = 109)**	**SufficientVitD** **status** **(*N* = 28)**	**LowVitD** **status** **(*N* = 81)**	***P* value**
**Obstetrical data**				
Multiple gestation	29 (27)	8 (28)	21 (26)	0.78
PPROM	21 (19)	5 (18)	16 (20)	0.82
Preeclampsia	22 (20)	5 (18)	17 (21)	0.79
MTR/PA	19 (17)	5 (18)	14 (17)	1.00
GD	4 (3.6)	0 (0)	4 (5)	0.57
Chorioamnionitis	25 (23)	5 (18)	20 (25)	0.60
Antenatal steroids	85 (78)	22 (78)	63 (78)	0.93
Mg^2+^ sulfate	81 (74)	23 (82)	58 (72)	0.27
Cesarean section	88 (81)	20 (71)	68 (84)	0.68
**Neonatal data**				
GA, mean (SD), weeks	26 (1.4)	26.5 (1.5)	26 (1.4)	0.11
BW, mean (SD), g	801 (212)	828 (198)	792 (217)	0.40
SGA	23 (21)	6 (21)	17 (21)	0.96
Female	60 (55)	19 (68)	41 (51)	0.11
5 min Apgar score <7	24 (22)	4 (14)	20 (25)	0.27
Placental transfusion	66 (60)	19 (68)	47 (58)	0.17
Digestive disease	8 (7)	3 (11)	5 (6)	0.42
Severe cerebral injury	10 (9)	2 (7)	8 (10)	1.00
Early onset sepsis	9 (8)	2 (7)	7 (9)	1.00
Late onset sepsis	56 (51)	12 (43)	44 (54)	0.29
Treatment for PDA	38 (35)	8 (28)	30 (37)	0.41
Severe ROP	12/103 (12)	2/26 (8)	10/77 (13)	0.72
BPD any grade	59/97 (61)	12/25 (48)	47/72 (65)	0.12
BPD stage 2 – 3	31/97 (32)	5/25 (20)	26/72 (36)	0.21
Death before discharge	12 (11)	3 (11)	9 (11)	1.00

*BPD, bronchopulmonary dysplasia; BW, birth weight (gr); GA, gestational age (weeks); GD, gestational diabetes mellitus; MTR/PA, metrorrhagia/placental abruption; PDA, patent ductus arteriosus; PPROM, preterm premature rupture of membranes; ROP, retinopathy of prematurity; SGA: small for gestational age; SufficientVitD status, infants with cord blood 25(OH)D levels > 75 nmol/L; LowVitD status, infants with cord blood 25(OH)D levels ≤ 75 nmol/L*.

**Table 2:** Early respiratory outcomes according to vitamin D status at birth.

**Characteristics, *n* (%)**	**All** **population** **(*N* = 109)**	**SufficientVitD** **status** **(*N* = 28)**	**LowVitD** **status** **(*N* = 81)**	***P* value**
Surfactant	83 (76)	21 (75)	62 (76)	0.87
Timing (min)*	50 (25–90)	62 (30–90)	41 (25–60)	0.09
FiO_2_ (%), mean (SD)	51 (22)	41 (18)	54 (22)	0.02
> 2 doses	19 (17)	3 (11)	16 (20)	0.39
LISA/INSURE procedure	46 (42)	14 (50)	32 (39)	0.33
MV W1	52 (48)	9 (32)	43 (53)	0.05
MV W1 duration (h)*	120 (24–168)	72 (15–144)	144 (14–168)	0.24
HFO	21 (40)	1 (11)	20 (46)	0.06
MV DR	24 (39)	4 (14)	20 (25)	0.30
MV DoL3	35 (32)	6 (21)	29/80 (36)	0.15
MV DoL7	28/106 (26)	2 (7)	26/78 (33)	0.007
Death or MV DoL7	31 (28)	2 (7)	29 (36)	0.003

*Median (IQR); HFO, high frequency oscillation; MV W1, mechanical ventilation in the first week of life; MV DR, mechanical ventilation in the first 15 min after birth (delivery room); MV DoL3, mechanical ventilation on day of life 3; MV DoL7, mechanical ventilation on day of life 7; SufficientVitD status, infants with cord blood 25(OH)D levels > 75 nmol/L; LowVitD status, infants with cord blood 25(OH)D levels ≤ 75 nmol/L.

In the multivariate analyses, and after backward elimination of variables, adjusted for GA, infants with LowVitD status had higher odds of death or being mechanically ventilated at DoL7 with an adjusted OR (95% CI) of 10.6 (1.13–65.1) (*p* < 0.05) (Table 3). The same association was found with the outcome of MV DoL7 with an adjusted (on GA) OR (95% CI) of 8.14 (1.37–48.5) (*p* <0.05).

**Table 3:** Regression analysis: factors associated with the combined outcome of death or mechanical ventilation on the 7th day after birth (death or MV DOL7).

	**Adjusted OR (95%IC)**	***P* value**
GA	0.39 (0.25–0.60)	<0.0001
LowVitD status	10.6 (1.13–65.1)	0.01

*GA, gestational age (weeks); LowVitD status, infants with cord blood 25(OH)D levels ≤ 75 nmol/L*.

**Figure 2:**
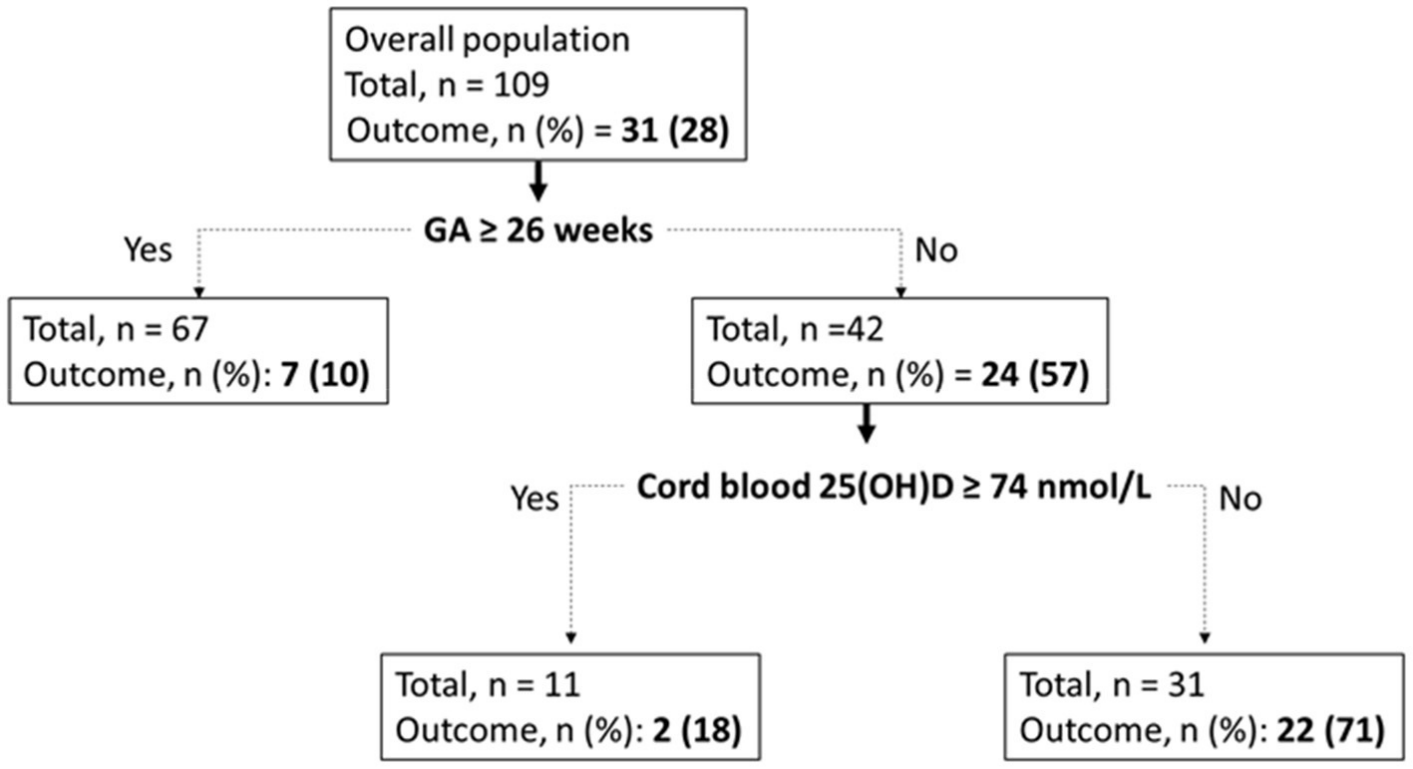
CART model for death or MV DoL7 outcome. In each node (rectangle), the category “Outcome” refers to the presence of the combined outcome of death or MV DoL7, with *n* and percentage of related infants.

The CART analysis revealed GA, with the threshold value at 26 weeks, and cord blood 25(OH)D levels <74 nmol/L were important variables to predict the combined outcome of death or VM DoL7 (Figure 2). The rate was 10% in infants with GA above 26 weeks and reached 71% in infants with GA <26 weeks who had cord blood 25(OH)D levels lower than 74 nmol/L. The mean accuracy of this algorithm was estimated to be equal to 0.77 CI 95% [0.64-0.89].

## Discussion

In this study, we found an association between vitamin D status at birth and early respiratory outcome in infants with GA <29 weeks. Infants who had low blood 25(OH)D levels ≤ 75 nmol/L at birth had early adverse outcomes with higher oxygen need for RDS and prolonged mechanical ventilation by the end of the first week of life compared to their counterparts that had adequate blood vitamin D levels.

Studies that evaluated the influence of vitamin D status on early respiratory outcome of very preterm infants have shown conflicted findings (15, 26–28). The makers used to define RDS severity and vitamin D deficiency varied between the studies. Some of them have found a high rate of RDS and markers of severe respiratory disease while others did not (15, 26–30). Onwuneme C et al. have found in a population of very preterm infants (GA <32 weeks) an association between vitamin D deficiency at birth [25(OH)D levels <30 nmol/L] and clinical signs of acute respiratory morbidity with higher oxygen needs and a greater rate of MV in the delivery room and during the neonatal period (15). We also found higher FiO_2_ levels at surfactant administration while the timing to administration tended to be shorter, and higher need of high frequency oscillation; these results can be considered marker of respiratory disease severity. In contrast to these previous studies, we included a population of more premature infants who are particularly vulnerable to a severe form of RDS with high needs of intubation and prolonged mechanical ventilation. We also included infants with higher prevalence (26%) of sufficient vitamin D concentrations (above 75 nmol/L); while this prevalence was usually reported <5–10%. Including infants with higher cord blood 25(OH)D levels may have revealed the adverse consequences of vitamin D deficiency in our study. Our study adds further evidence on the influence of vitamin D status at birth on early respiratory outcomes in preterm infants.

Although we used the international vitamin D definition to classify infants, the CART analysis identified optimal cutoff points for 25(OH)D of 74 nmol/L, which is the threshold level commonly used in pediatric and adult medicine to define vitamin D sufficiency (20). In this method, the more important variables are ranked early on the classification tree, showing the important influence of vitamin D on early respiratory outcome, especially in infants with GA <26 weeks; the rate of death or MV DoL7 fourfold increased between SufficientVitD infants and their counterpart infants who had low 25(OH)D levels (*p* < 0.05). While the adequate vitamin D status is unknown in preterm infants; our study provides useful insights on the optimal 25(OH)D levels at birth.

Recent evidence suggests a biological role of vitamin D in normal lung development and maturation (2, 4, 30, 31). Vitamin D also contributes to surfactant synthesis and secretion by alveolar type II cells (31). The fetus is exclusively dependent on maternal vitamin D status, and transfer from the mother mainly occurs during the third trimester. The umbilical cord blood 25(OH)D level at birth is therefore representative of fetal vitamin D stores. Endothelial and alveolar type II cells express a nuclear receptor specific for the bioactive form of vitamin D [1,25 (OH)_2_D_3_], named vitamin D receptor (VDR). VDR response elements are also present in a subset of more than 500 genes involved in lung development in humans and rodents (32, 33). In animals, maternal gestational vitamin D depletion impairs fetal lung development with reduced alveolar numbers, decreased vascular density, altered pneumocyte type II differentiation, decreased tracheal size, and increased airway smooth muscle mass (2, 31). In the neonatal lungs of vitamin D-depleted rats, genes involved in the inflammatory response and vascular development, such as VEGF, have been found to be up- and downregulated, respectively (34). These functional and structural alterations are responsible for increased neonatal mortality with lower oxygen saturation, reported in preterm pups (35). In the long term, the offspring displayed increased airway resistance and low tidal volume (2). Some of these functional alterations, including frequent wheezing events, asthma, lung dysfunctions and respiratory tract infections, were also observed in children of vitamin D-deficient mothers (7–9). Finally, in rodents, maternal gestational vitamin D administration prevents neonatal mortality and lung injury induced by intrauterine exposure to endotoxin, which is a model of bronchopulmonary dysplasia (36). Based on these preclinical data, we can hypothesize that high oxygen and sustained MV needs for RDS observed in LowVitD status preterm infants result from delayed lung maturation.

This study had some intrinsic limitations due to the study design. Some commonly used markers of RDS severity such as respiratory severity score and ventilator settings are lacking; but high initial FiO_2_ levels, prolonged mechanical ventilation needs or frequently use of high frequency oscillation could be considered surrogate marker of RDS severity. Our sample size was too small to investigate the influence of vitamin D status at birth on other neonatal outcomes, especially BPD. Moreover, although our single-center experience may have limited bias linked to differences in health care practices, further multicenter studies with sufficient size are needed to confirm our findings.

In conclusion, in this study, we found that preterm infants with GA <29 weeks that had 25(OH)D levels at birth ≤ 75 nmol/L had early adverse outcomes. Our findings provide additional evidence for the important role of fetal vitamin D on lung development and provide new insights into optimal 25(OH)D levels. In very and extremely preterm infants early vitamin D status, and thus maternal vitamin D status, can be considered marker of respiratory disease including RDS and BPD. Early respiratory outcomes could be improved by preventing maternal gestational vitamin D deficiency. Larger studies should be performed to confirm these findings and to investigate the influence of vitamin D on other neonatal outcomes and especially on long-term respiratory functions.

## Data Availability Statement

The raw data supporting the conclusions of this article will be made available by the authors, without undue reservation.

## Ethics Statement

The studies involving human participants were reviewed and approved by Assistance Publique des Hôpitaux de Marseille, Comission Informatique et Liberté. Written informed consent from the participants' legal guardian/next of kin was not required to participate in this study in accordance with the national legislation and the institutional requirements.

## Author Contributions

HP and AS collected data, drafted the initial manuscript, and reviewed and revised the manuscript. EG collected data and reviewed and revised the manuscript. CB collected data, carried out vitamin D analyses, and reviewed and revised the manuscript. CR designed the data collection instruments and reviewed and revised the manuscript. J-FL participated to analysis and interpretation of data and critically reviewed the manuscript for important intellectual content. VP designed the data collection instruments and carried out the statistical analyses. FB conceptualized and designed the study and reviewed and revised the manuscript. All authors approved the final manuscript as submitted and agreed to be accountable for all aspects of the work.

## Conflict of Interest

The authors declare that the research was conducted in the absence of any commercial or financial relationships that could be construed as a potential conflict of interest.

## Publisher's Note

All claims expressed in this article are solely those of the authors and do not necessarily represent those of their affiliated organizations, or those of the publisher, the editors and the reviewers. Any product that may be evaluated in this article, or claim that may be made by its manufacturer, is not guaranteed or endorsed by the publisher.
