# Supplementary material for: Low Vitamin D Levels at Birth and Early Respiratory Outcome in Infants With Gestational Age Less Than 29 Weeks
Source: Front Pediatr. 2022 Jan 21;9:790839. doi: 10.3389/fped.2021.790839 (PMC8814585; doi:10.3389/fped.2021.790839)
Supplement: Supplementary file 1 [file Data_Sheet_1.docx]

**Supplementary Figure : Flow diagram**


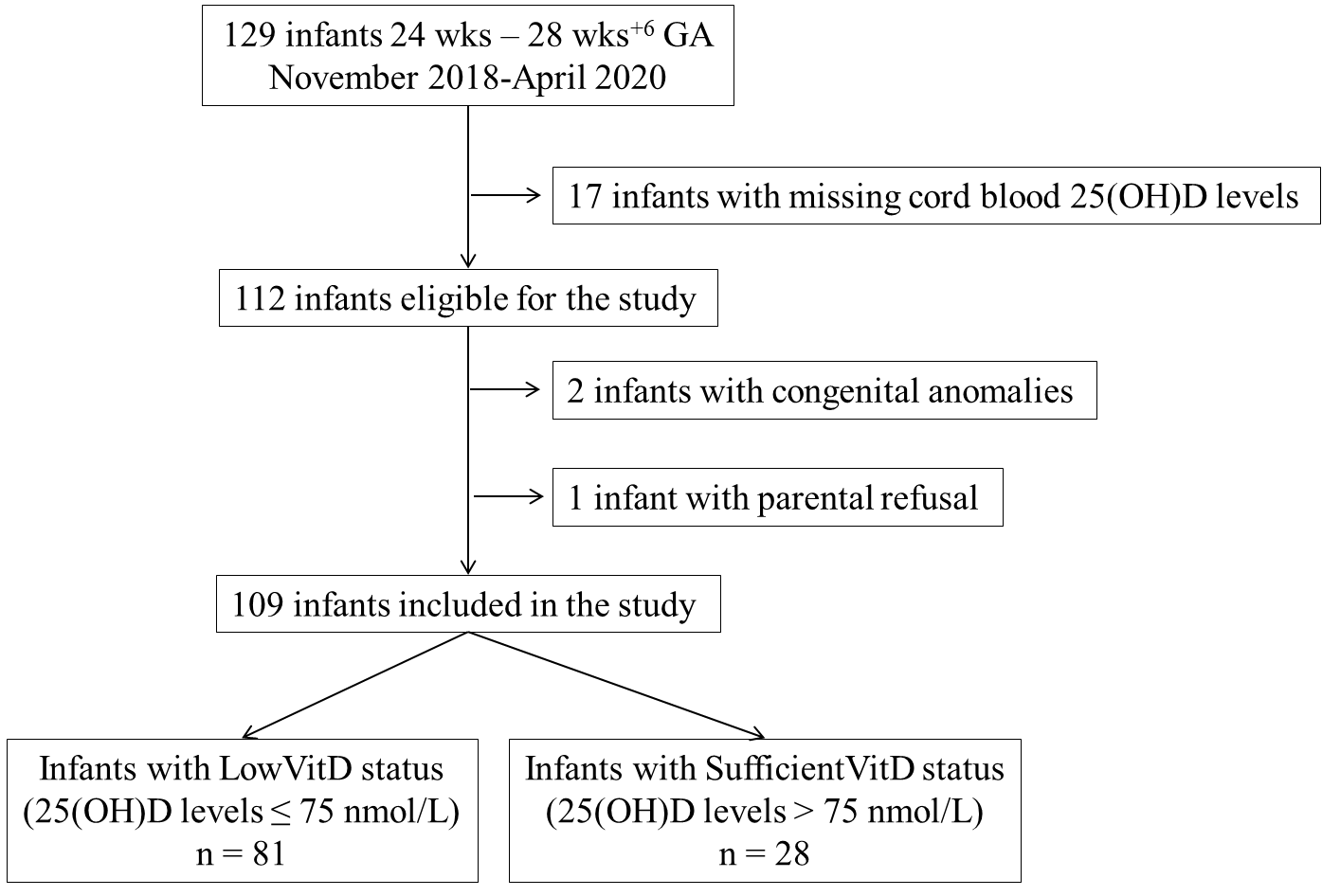


**Supplementary Table**

| Perinatal Characteristics | Death or MV DoL7 | No Death or MV DoL7 | *P* value |
| --- | --- | --- | --- |
| Multiple gestation, n (%) | 11 (35) | 18 (23) | 0.18 |
| PPROM, n (%) | 8 (26) | 13 (17) | 0.27 |
| Preeclampsia, n (%) | 4 (13) | 17 (22) | 0.42 |
| MTR/PA, n (%) | 5 (16) | 14 (18) | 1.00 |
| GD, n (%) | 1 (3.2) | 3 (3.8) | 1.00 |
| Chorioamnionitis, n (%) | 10 (32) | 15 (19) | 0.11 |
| Antenatal steroids, n (%) | 28 (90) | 73 (93) | 0.68 |
| Mg^2+^ Sulfate, n (%) | 21 (68) | 60 (77) | 0.32 |
| Cesarean section, n (%) | 20 (64) | 61 (78) | 0.14 |
| GA (g), mean (SD) | 25 (1) | 26 (1.3) | <0.0001 |
| BW (weeks) mean (SD) | 676 (187) | 850 (202) | <0.0001 |
| SGA, n (%) | 6 (19) | 17 (22) | 0.78 |
| Female, n (%) | 17 (55) | 44 (56) | 0.88 |
| 5 min Apgar score < 7, n (%) | 13 (41) | 11 (14) | 0.001 |
| Placental transfusion, n (%) | 13 (41) | 53 (68) | 0.01 |
| Surfactant, n (%) | 29 (93) | 54 (69) | 0.006 |
| 25(OH)D nmol/L, mean (SD) | 40 (23) | 58 (34) | 0.002 |
| LowVitD status, n (%) | 29 (93) | 52 (67) | 0.003 |

BPD: bronchopulmonary dysplasia; BW: birth weight (gr); GA: gestational age (weeks); GD: gestational diabetes mellitus; MTR/PA: metrorrhagia/placental abruption; PDA: patent ductus arteriosus; PPROM: preterm premature rupture of membranes; SGA: small for gestational age; LowVitD status: infants with cord blood 25(OH)D levels < 75 nmol/L.
